# Absence of evidence or evidence of absence: reflecting on therapeutic implementations of attentional bias modification

**DOI:** 10.1186/1471-244X-14-8

**Published:** 2014-01-15

**Authors:** Patrick JF Clarke, Lies Notebaert, Colin MacLeod

**Affiliations:** 1 School of Psychology, University of Western Australia, Crawley 6009, Western Australia, Australia; 2 School of Psychology, Babes-Bolyai University, Strada Mihail Kogãlniceanu 1, Mihail Kogalniceanu St, Cluj-Napoca 3400, Romania

**Keywords:** Attentional bias modification, ABM, Cognitive bias modification, CBM, Experimental psychopathology

## Abstract

Attentional bias modification (ABM) represents one of a number of cognitive bias modification techniques which are beginning to show promise as therapeutic interventions for emotional pathology. Numerous studies with both clinical and non-clinical populations have now demonstrated that ABM can reduce emotional vulnerability. However, some recent studies have failed to achieve change in either selective attention or emotional vulnerability using ABM methodologies, including a recent randomised controlled trial by Carlbring et al. Some have sought to represent such absence of evidence as a sound basis not to further pursue ABM as an online intervention. While these findings obviously raise questions about the specific conditions under which ABM procedures will produce therapeutic benefits, we suggest that the failure of some studies to modify selective attention does not challenge the theoretical and empirical basis of ABM. The present paper seeks to put these ABM failures in perspective within the broader context of attentional bias modification research. In doing so it is apparent that the current findings and future prospects of ABM are in fact very promising, suggesting that more research in this area is warranted, not less.

## Background

Attentional bias modification (ABM) is an emerging treatment approach designed to alter patterns of attentional selectivity favouring the processing of threatening information that are implicated in the development and maintenance of psychopathology, most notably anxiety disorders. The last 5 years has seen a rapid expansion in the volume of research examining the therapeutic benefits of ABM. On balance, results have been very encouraging, with more than 20 studies now demonstrating that the modification of selective attention for threat can reduce levels of anxiety vulnerability [1]. In addition to these positive results, some recent studies have failed to demonstrate clinical benefits under specific conditions. One of the most recent of these was the randomised control trial by Carlbring and colleagues [2] which failed to modify biased attention and anxiety symptoms among a group of socially anxious individuals. While the old scientific truism reminds us not to mistake absence of evidence as evidence of absence, a recent review of ABM research appears to have made such a mistake in suggesting that current empirical findings provide reason to believe that attentional bias modification has little potential as a therapeutic tool. This brief review by Emmelkamp [3] draws inspiration from the tale of the Emperor’s New Clothes. In this story, despite the total absence of objective evidence supporting the existence of the garment, the protagonist, for socially motivated reasons, chooses to act as if such evidence existed. Emmelkamp’s review suggests that this same phenomenon may apply to those who profess to believe the validity of empirical findings regarding the clinical benefits of attentional bias modification, who choose to do so despite the absence of objective evidence. Indeed, Emmelkamp goes so far as to conclude that that the weight of current empirical evidence suggests that “…there is no need yet to investigate the implementation of attentional bias modification through the internet” (p1). In making this suggestion we believe that the review is perhaps showing more similarity with an alternative true tale, originating in the 1801 Battle of Copenhagen featuring one Vice-Admiral Horatio Nelson. It is said that upon receiving a signal to withdraw his ships, which he did not wish to acknowledge, Nelson, in an act of motivated denial of objectively identifiable evidence, raised his telescope to his one blinded eye, and commented “I really do not see the signal”, thus coining the phrase ‘turning a blind eye’. While we do not believe that Emmelkamp’s recent commentary on ABM has sought to engage in the same motivated denial of current evidence as Nelson, it seems that on the issue of empirical support for ABM, not only has this been observed through an overly narrow scope, but the scope may have been selectively directed, and the resulting vision compromised to an extent that limits the voracity of the conclusions concerning the objective state of the field. The following seeks to briefly consider recent findings within the context of the emergence of ABM from experimental psychopathology, reviews the current evidence regarding the therapeutic potential of ABM, and considers whether recent failed ABM implementations provides adequate reason to abandon ABM as a potential clinical intervention.

### The emergence of ABM from experimental psychopathology

Attentional bias modification can be considered a product of the relatively new field of psychological research broadly termed experimental psychopathology. The principal goal of experimental psychopathology has been to establish the low level patterns of information processing (such as attention and interpretation) that contribute to emotional pathology. More recently however, researchers in this field have sought to develop cognitive-experimental technologies capable of modifying biased patterns of information processing to produce clinical benefits. Some have expressed frustration that two decades of experimental psychotherapy research has resulted in little clinical application [3], with cognitive bias modification (including ABM) emerging as one of the few promising products of these enquiries. While we sympathise with such frustration (scientific progress being almost invariably slow), it is important to consider the emergence of this in the context of the development of previous treatment approaches. A backward glance reminds us that more than half a century elapsed between Pavlov spotting the ‘conditioned reflex’ in 1901 and formalisation of behavioural treatment approaches based on systematic desensitisation in the 1950s [4]. It would then be another half century until innovative researchers, such as Emmelkamp himself, sought to adapt such treatments to permit online delivery (the creation of the internet being a necessary precursor). Such a historical context highlights that, by comparison, the emergence of cognitive bias modification techniques from experimental psychopathology appears to have occurred at blistering speed.

It is scarcely a decade since MacLeod and colleagues [5] provided the first experimental demonstration that attentional bias for threatening material can be altered, and that this exerts a direct impact on emotional vulnerability. The first work delivering such ABM to clinical samples began only in the last five years and has expanded rapidly [6]. Three early studies provided compelling evidence that ABM can attenuate the clinical symptoms of patients suffering from anxiety disorders. In an RCT, Schmidt et al. [7] found that 72% of individuals diagnosed with social anxiety disorder who completed eight ABM sessions across 4 weeks no longer met clinician-rated diagnostic criteria for the disorder post-treatment, compared to 12% in a non-training control condition, with gains being maintained at four-month follow-up. In a similar 4 week design, Amir et al. [8] also found that, at post-treatment, 50% of those who received ABM no longer met diagnostic criteria for social anxiety disorder, compared to 14% in the control condition. A third study involving patients with generalised anxiety disorder, conducted by Amir et al. [9], found that eight sessions of ABM across four weeks resulted in 50% of those receiving ABM no longer meeting diagnostic criteria, compared to 13% of participants exposed a non-training control condition. Subsequent studies have also shown promising results in paediatric anxiety disorders using home-based delivery of ABM. Waters et al. [10] revealed that two weeks (12 sessions) of ABM completed by children with clinical anxiety at home resulted in 50% of those in active training no longer meeting diagnostic criteria compared to 8% in the control condition. Others still have demonstration that ABM can reduce risk factors for recurrent depressive episodes among vulnerable individuals as evidenced by reduction in self-reported depressions symptoms and objective indices of risk such as cortisol awakening response [11]. By most standards, the results of these studies could be considered a very encouraging initial foray of ABM into clinical settings, and these represent only a fraction of the research which has demonstrated that the modification of selective attention can have positive emotional consequences [12]. Nevertheless it is important to consider such findings within the context of the broader ABM literature.

### Meta-analytic findings of ABM and their limitations

At least two meta-analyses have now been conducted providing some general insight as to the therapeutic potential of ABM [12,13]. While these can be a useful tool for examining the broad evidence in relation to ABM, as with all meta-analyses, by combining the data of various studies critical distinctions can be missed. Perhaps most notably, the Hallion and Ruscio meta-analysis [13] has a number of serious limitations. Not only did this study pool the results of both attentional bias modification and interpretive bias modification studies, the examination of the impact of bias modification was compromised by inappropriately combining emotional measures. Specifically, they combined emotional effects measured at time points where there is no expectation that bias modification will impact emotion (immediately after cognitive bias modification but before an emotional stressor in single session implementations) with time points where it is expected that bias modification will have an emotional impact (post-treatment in multi-session implementations), thereby underestimating therapeutic emotional effects and over-estimating spurious mood effects following the bias modification tasks. This paper was also highly inclusive of bias modification studies (e.g. including those with dubious ABM methods [14]) and handled missing effect sizes by imputing values of 0.00. Thus, it is somewhat unsurprising that estimates of effect sizes across the pooled attentional and interpretive bias modification studies were small (*g* = 0.13 – 0.29).

The meta-analysis by Hakamata, et al. [12] by comparison includes only studies with computable effect sizes and focuses exclusively on attentional bias modification, providing more relevant information on ABM. This paper put overall estimated effect sizes in the medium range (*d* = 0.61). Furthermore, it indicated that these effects seem to be larger amongst patient populations (*d* = 0.78) as compared to non-patients (*d* = 0.48). Such clinical effect sizes begin to compare favourably with traditional psychological interventions (e.g. *d* = 0.86; [15]). This seems contrary to the claim in Emmelkamp’s recent review [3] that there “is not robust evidence that attention training is of clinical value”. Indeed, when one considers that effect sizes for CBT are likely to be enhanced by poorly-matched control conditions [16] as compared to the tightly matched controls in ABM, such interventions appear to have considerable promise. It is important to note however that the studies included in these meta-analyses do not meet the stringent criteria of randomised control trials, and indeed, only a minority of ABM studies conducted to date do. These studies should therefore be interpreted with caution. Furthermore, a limitation with both these meta-analyses is that the increasing volume of ABM research means that they have quickly become dated and do not consider a number of recent findings that have, and have not succeeded in implementing ABM. Thus, despite these generally encouraging findings it is important to consider these more recent studies and their broader implications for attentional bias modification as an emerging treatment approach.

### What do recent failures suggest about the potential effectiveness of ABM?

At the time of its publication, the study by Carlbring et al. [2] represented the first study to have implemented an ABM task without therapeutic success within a controlled trial. There have since been a been a number of other studies that have delivered intended attentional bias modification tasks that have neither successfully modified biased attention or impacted clinical symptomology. Several of these have targeted social anxiety disorder [17-20], with another unsuccessfully attempting to modify biased attention in sufferers of posttraumatic stress disorder [21]. What has tended to be overlooked in a number of these studies (e.g. [2]) and within recent reviews of this work [3], but which is critical to note, is that none of these studies succeeded in modifying attentional bias. That is, it is not the case that these studies altered patterns of biased attention but this failed to influence measures of emotional vulnerability. Rather, they simply failed to alter patterns of selective attention that are known to causally underpin emotional vulnerability. Without such a change in attention, a change in emotion therefore cannot be expected. Hence, these studies represent manipulation failures, not evidence against the potential therapeutic value of attentional bias modification. To conclude that attentional bias modification is therapeutically ineffective on the basis of studies that failed to modify attentional bias would be akin to drawing the conclusion that the surgical removal of tumours is an ineffective treatment for cancer on the basis of studies that performed surgery but failed to remove any tumours. In each case a procedure has been performed with the intention of altering a specific target, but the critical target of the intervention was not achieved. These studies therefore represent an absence of evidence for the effectiveness of ABM, but not evidence that ABM is ineffective.

Indeed, the very fact that the studies which have failed to modify selective attention have also failed to modify emotional vulnerability provides reassurance that the theoretical basis for ABM is sound. Specifically, it highlights that when a task achieves its goal of successfully altering attention, it will reliably produce emotional change, and when it doesn’t, it won’t. This pattern of effects holds true more generally across the literature. We compiled a list of 42 ABM studies (39 papers) conducted to date (see Additional file 1), of which 29 include measures of both attentional bias change and emotional vulnerability, and are therefore able to inform the link between this proposed causal mechanism and its emotional impact. Of these 29 studies, 26 are entirely consistent with this link in that when attentional bias modification is achieved so is the consequent change in emotional vulnerability (n = 16), and when attentional bias modification is not achieved, no impact on emotional vulnerability is observed (n = 10). Of the three remaining studies, one successfully modified attentional bias but observed an impact only on behavioural measures (willingness to approach a feared stimulus) [22], and two focused on specific phobias [23,24] which, as noted elsewhere [6], may be resistant to emotional change via cognitive bias modification techniques. Of the 16 studies that succeeded in modifying both attention and emotional vulnerability four were conducted with clinical samples (six sub-clinical), while for those that did not succeed in modifying attentional bias, seven were conducted with clinical samples (one sub-clinical). The failure of a number of studies to modify selective attention should certainly invite empirical scrutiny into the precise task conditions and modes of delivery that are most likely to be effective in producing a bias change. However, they provide little reason to conclude, as Emmelkamp does [3], that ABM procedures and the various modalities in which they may be delivered (e.g. online, portable device) should not be pursued as a promising form of therapeutic intervention for clinical populations, as the research clearly tells us that when the modification of attentional bias is achieved, emotional benefits follow.

### Could the positive results of ABM be due to demand effects?

In his recent review Emmelkamp [3] suggests that expectancy and demand effects are likely to play a role in producing positive emotional effects in ABM. The potential influence of demand and expectancy on treatment outcome is an issue of concern for a range of interventions so it is worth considering whether such extraneous participant factors could potentially carry effects derived from ABM interventions. It is well known that the effects of cognitive and behavioural therapeutic interventions may benefit from poorly-matched control conditions (such as waitlist) where the comparative effects from active treatment conditions may be enhanced by expectancy, demand, and non-specific treatment factors which are absent in the control group [16]. By comparison, ABM resists demand and expectancy effects far more effectively than the great majority of CBT interventions due to the quality of the control condition. In most ABM tasks the control condition is tightly matched with the treatment condition in virtually all parameters, except the training contingency. For example, in the attentional probe approach (e.g. [25]), both active ABM and control conditions present the same stimuli, delivered at the same time, for the same duration and require the same response. The only difference between the active ABM and control condition is the presence of a contingency between the probe location and the stimulus type in the active ABM condition (with probes always appearing in the location of non-threatening stimuli to encourage an attentional bias away from threat), which is absent in the control condition. The subtlety of this difference between control and active ABM conditions means that researchers can confidently rule out the influence of non-specific factors as potential therapeutic agents (e.g. such as stimulus exposure). Also, because of this small difference between conditions, participants consequently are likely to have no awareness of whether or not they are in fact receiving an active intervention, which means there is little capacity for demand or expectancy effects to selectively influence the treatment condition and not the control condition. When ABM researchers have sought to examine participant awareness of their allocation to treatment conditions they have found that most (79% of those in the control condition and 72% of those in the ABM condition) believe that they are not receiving an active treatment at all, with no significant differences in this awareness between conditions [8].

While Emmelkamp’s review proposes that ABM findings may be due to demand effects it provides no suggestions about how such effects could occur. Indeed, when one considers how demand and expectancy could contribute to the effects observed in ABM it becomes apparent that this would have to be so highly systematic as to stretch credulity. Participants would need to be aware of the purpose of ABM tasks and feign the acquisition of an attentional bias only in the active ABM condition and not the control. Even if this were plausible, the review also fails to engage with the vexing issue of how such demand characteristics happen to produce emotional benefits in only those studies that successfully modify selective attention and not in those which have failed to do so. Thus, it seems that the burden of demand and expectancy effects does not weigh heavily on ABM research, but instead falls upon studies that involve comparison of active psychotherapeutic treatments and waitlist controls. This includes the type of internet-based treatment promoted in Emmelkamp’s recent review as one of the key reasons that researchers do not need to investigate online delivery of ABM [26].

### Is there any need for attentional bias modification as an intervention?

Perhaps the most provocative element of Emmelkamp’s recent review [3] is the insistence that current evidence suggests “…there is no need yet to *investigate* the implementation of attentional bias modification through the internet” (p1, emphasis added). It seems that despite clear evidence that altering biased attention for threat can reduce emotional vulnerability, and encouraging results with clinical samples, the failure of a number of studies to modify selective attention should be grounds for all researchers to cease investigations into the clinical applications of attentional bias modification using the internet. By way of justification, Emmelkamp cites findings of internet-delivered treatment for social anxiety from his own research group. This study certainly produced very respectable between-group effect sizes (d = 0.86) for those receiving active treatment. However, the implication that the efficacy of an existing intervention means that promising alternative and/or adjunct treatment approaches need not be investigated is a curious one, quite antithetical to the cycle of innovation, refinement, and improvement that characterises scientific progress. It also ignores the potential advantages of ABM interventions in terms of their simplicity and brevity compared to ‘brief’ CBT approaches taking 6–12 weeks incorporating psycho-education, cognitive restructuring, exposure and behavioural experiment components. In contrast, two 15 minute ABM sessions a week for four weeks have been shown to achieve within-group treatment effect sizes of up to d = 1.92 for social anxiety disorder [8] and d = 1.40 for generalised anxiety disorder [9] with follow-up data suggesting that benefits are maintained over time. Hence, it is difficult to understand the basis for suggesting that the existence of effective online interventions should preclude researchers from investigating what is a promising alternative treatment approach. The fact that that patterns of biased information known to characterise anxiety pathology can be directly targeted by computer-based ABM tasks that can be delivered remotely suggests such interventions hold significant potential as either a stand-alone or complimentary intervention for anxiety disorders that may be ideally suited to online delivery. Thus, unlike Emmelkamp, we believe that the current evidence provides compelling reasons to investigate online delivery of ABM, and we would encourage more research in this area, not less.

### Future research

On one issue we believe we are in complete agreement with Emmelkamp. That is, ABM is not ready for wide-scale dissemination as a mainstream intervention, nor should it yet be directly marketed to mental health consumers. The number of therapeutic successes observed provides a tantalising demonstration of what ABM is capable of, while the failures to consistently modify biased attention for threat highlights the need for more reliable means of achieving change in the target cognitive process. Thus, as is the case for any fledgling psychotherapeutic intervention, an early objective for ABM researchers must be to optimise capacity to modify the target process; namely, biased attentional response to negative information. The observation that the magnitude of attentional change elicited by ABM treatment predicts the magnitude of change in emotional symptomology [12] suggests that as ABM tasks get better at achieving attentional change, so too will they become more effective in alleviating emotional dysfunction. All of the findings reviewed here have utilised variants of the attentional probe task originally developed by MacLeod et al. [5]. However, it is highly improbable that the first ABM task ever developed will prove to be the best at producing robust and enduring attentional change. As researchers seek to refine and enhance current ABM tasks and develop innovative new approaches, we can expect to see progressive improvement in their attentional impact. In this regard we can be confident that ABM has not peaked. Such future research will likely shed light on the reasons why particular studies have failed to alter selective attention, whereas others have succeeded. For example, it would be interesting to investigate whether participants’ belief that they are not receiving active treatment (when they are in fact being delivered ABM) reduces task adherence and the attentional and emotional benefits of ABM. While the formality of a lab environment may contribute to task engagement regardless of beliefs concerning treatment condition, being convinced that one is not receiving treatment could have greater consequence for motivation and engagement among those who complete ABM tasks at home. If this is indeed the case, it would be critical for future studies to carefully consider the type of instructions that participants receive to ensure that task engagement is not compromised. Additionally, future research could usefully seek to establish how ABM may best be incorporated with traditional therapeutic approaches to enhance clinical outcomes. This will help to identify whether ABM may best be used as a precursor to psychotherapy, delivered simultaneously with other interventions, or if it can assist in the maintenance of gains and prevention of relapse via administration post-therapy.

## Conclusions

ABM has produced many positive findings that confirm its capacity to attenuate emotional vulnerability in non-clinical samples and to reduce the symptoms of emotional dysfunction in clinical patients. The fact that some studies have failed to successfully modify selective attention, and consequently have not modified emotional vulnerability, suggests that more work remains to be done in order to identify the conditions under which ABM is likely to be most effective in producing the target attentional change. This observation underscores the importance of increasing, not reducing research into ABM approaches. Our knowledge of the precise conditions under which exposure-based treatments can effectively reduce anxiety has come a long way in the century since Pavlov first began ringing bells and collecting saliva. The early and continuing successes of ABM approaches suggest that this and other cognitive bias modification techniques are likely to have a bright future. Undoubtedly, the attainment of this bright future will depend upon the investment of sustained and creative research effort. One would be well advised to watch this space rather than turn a blind eye.

## Competing interests

The authors have no competing interests to declare.

## Authors’ contributions

All authors contributed to this manuscript with PC taking major responsibility for the content. All authors read and approved the final manuscript.

## Pre-publication history

The pre-publication history for this paper can be accessed here:

http://www.biomedcentral.com/1471-244X/14/8/prepub

## Supplementary Material

Additional file 1 Studies that have and have not succeeded in modifying attentional bias, and the consequent presence and absence of emotional effects. Click here for file
