# Supplementary material for: Absence of evidence or evidence of absence: reflecting on therapeutic implementations of attentional bias modification
Source: BMC Psychiatry. 2014 Jan 15;14:8. doi: 10.1186/1471-244X-14-8 (PMC3899426; doi:10.1186/1471-244X-14-8)
Supplement: Additional file 1 — Studies that have and have not succeeded in modifying attentional bias, and the consequent presence and absence of emotional effects. [file 1471-244X-14-8-S1.docx]

Studies that have and have not succeeded in modifying attentional bias, and the consequent presence and absence of emotional effects

| **Authors** | **Sample** | **No. ABM sessions** | **Bias Change Assessed** | **Emotional Impact Assessed** | **Significant bias change** | **Significant emotional impact** |
| --- | --- | --- | --- | --- | --- | --- |
| Amir, N. et al. [[1](#_ENREF_1)] (2009) | Clinical - SAD | 8 | Y | Y | Y | Y |
| Eldar, et al. [[2](#_ENREF_2)] (2012) | Clinical - Pediatric Anxiety | 4 | Y | Y | Y | Y |
| Waters, et al. [[3](#_ENREF_3)] (2013) | Clinical - Pediatric Anxiety | 12 | Y | Y | Y | Y |
| Amir, N., et al. [[4](#_ENREF_4)] (2009) | Clinical - GAD | 8 | Y | Y | Y | Y |
| Hazen, et al. [[5](#_ENREF_5)] (2009) | Sub-clinical - High worry | 5 | Y | Y | Y | Y |
| Hayes, et al. [[6](#_ENREF_6)] (2010) | Sub-clinical - High worry | 1 | Y | Y | Y | Y |
| Bar-Haim, et al. [[7](#_ENREF_7)] (2011) | Sub-clinical - High trait anxious | 2 | Y | Y | Y | Y |
| Li, S., et al. [[8](#_ENREF_8)] (2008) | Sub-clinical - High Social Anxiety | 7 | Y | Y | Y | Y |
| Amir, et al. [[9](#_ENREF_9)] (2008) | Sub-clinical - High Social Anxiety | 1 | Y | Y | Y | Y |
| Browning, et al. [[10](#_ENREF_10)] (2012) | Sub-clinical - Depression history | 28 | Y | Y | Y | Y |
| Eldar, et al. [[11](#_ENREF_11)] (2008) | Non-clinical | 1 | Y | Y | Y | Y |
| Krebs, et al. [[12](#_ENREF_12)] (2010) | Non-clinical | 1 | Y | Y | Y | Y |
| Hirsch, et al. [[13](#_ENREF_13)] (2011) | Non-clinical | 1 | Y | Y | Y | Y |
| MacLeod, et al. [[14](#_ENREF_14)] (2002) - Expt 1 | Non-clinical | 1 | Y | Y | Y | Y (p < .06) |
| MacLeod, et al. [[14](#_ENREF_14)] (2002) - Expt 2 | Non-clinical | 1 | Y | Y | Y | Y |
| Dandeneau, & Baldwin [[15](#_ENREF_15)](2009) | Non-clinical | 1 | Y | Y | Y | Y |
| Britton, et al. [[16](#_ENREF_16)] (2013) | Clinical - Pediatric Anxiety | 5.7 (mean) | Y | Y | N | N |
| Schoorl, et al. [[17](#_ENREF_17)] (2013) | Clinical - PTSD | 8 | Y | Y | N | N |
| Boettcher, et al. [[18](#_ENREF_18)] (2013) | Clinical - SAD | 14 | Y | Y | N | N |
| Boettcher, et al. [[19](#_ENREF_19)] (2012) | Clinical - SAD | 11 | Y | Y | N | N |
| Carlbring, et al. [[20](#_ENREF_20)] (2012) | Clinical - SAD | 8 | Y | Y | N | N |
| Neubauer, et al. [[21](#_ENREF_21)] (2013) | Clinical - SAD | 8 | Y | Y | N | N |
| Rapee, et al. [[22](#_ENREF_22)] (2013) | Clinical - SAD | 39 (med) | Y | Y | N | N |
| Kruijt, et al. [[23](#_ENREF_23)] (2013) | Sub-clinical - High Depression | 1 | Y | Y | N | N |
| Klumpp, & Amir [[24](#_ENREF_24)] (2010) | Non-clinical | 1 | Y | Y | N | N |
| White, et al. [[25](#_ENREF_25)] (2011) | Non-clinical | 1 | Y | Y | N | N |
| **Authors** | **Sample** | **No. ABM sessions** | **Bias Change Assessed** | **Emotional Impact Assessed** | **Significant bias change** | **Significant emotional impact** |
| Van Bockstaele, et al. [[26](#_ENREF_26)] (2011) | Non-clinical | 1 | Y | Y | Y | N |
| Najmi & Amir [[27](#_ENREF_27)] (2010) | Sub-clinical - OC symptoms | 1 | Y | Y | Y | N |
| Reese, et al. [[28](#_ENREF_28)] (2010) | Sub-clinical - Spider fearful | 1 | Y | Y | Y | N |
| Dandeneau, et al. [[29](#_ENREF_29)] (2007) - Expt 3a | Non-clinical | 5 | N | Y | ? | Y |
| Dandeneau, et al. [[29](#_ENREF_29)] (2007) - Expt 3b | Non-clinical | 7 | N | Y | ? | Y |
| Bunnell, et al. [[30](#_ENREF_30)] (in press) | Clinical - SAD | 8 | N | Y | ? | N |
| Dandeneau, & Baldwin [[31](#_ENREF_31)] (2004) | Non-clinical | 1 | Y | N | Y | ? |
| Koster, et al. [[32](#_ENREF_32)] (2010) | Non-clinical | 1 | Y | N | Y | ? |
| MacLeod, et al. [[33](#_ENREF_33)] (2007) | Non-clinical | 1 | Y | N | Y | ? |
| Dandeneau, et al. [[29](#_ENREF_29)] (2007)Expt 2b | Non-clinical | 1 | Y | N*^a^* | Y | ? |
| Eldar & Bar-Haim [[34](#_ENREF_34)] (2010) | Non-clinical | 1 | Y | N*^a^* | Y | ? |
| Browning, et al. [[35](#_ENREF_35)] (2010) | Non-clinical | 1 | Y | N*^a^* | Y | ? |
| Blaut, et al. [[36](#_ENREF_36)] (2013) | Non-clinical | 1 | Y | N*^a^* | Y | ? |
| Brosan, et al. [[37](#_ENREF_37)] (2011) | Clinical - Anxiety outpatient | 4 | Y | Y*^b^* | Y | ? |
| Taylor, et al. [[38](#_ENREF_38)] (2011) | Non-clinical | 1 | Y | Y*^b^* | Y | ? |
| Suway, et al. [[39](#_ENREF_39)] (2013) | Non-clinical | 1 | Y | Y*^c^* | N | Y |

SAD - Social anxiety disorder; GAD – Generalised anxiety disorder; OC – Obsessive-compulsive.

*^a^*Impact of ABM on emotion not assessed as did not include an emotional stressor following the single-session ABM.

*^b^*Included a single group so not between-group comparison possible.

*^c^*Withing group change in attentional bias observed in active training but no between group change

**References**

1. Amir, N., et al., *Attention training in individuals with generalized social phobia: A randomized controlled trial.* Journal of Consulting and Clinical Psychology, 2009. **77**(5): p. 961-973.

2. Eldar, S., et al., *Attention bias modification treatment for pediatric anxiety disorders: a randomized controlled trial.* Am J Psychiatry, 2012. **169**(2): p. 213-20.

3. Waters, A.M., et al., *Attention training towards positive stimuli in clinically anxious children.* Developmental Cognitive Neuroscience, 2013. **4**: p. 77-84.

4. Amir, N., et al., *Attention modification program in individuals with generalized anxiety disorder.* Journal of Abnormal Psychology, 2009. **118**(1): p. 28-33.

5. Hazen, R.A., M.W. Vasey, and N.B. Schmidt, *Attentional retraining: A randomized clinical trial for pathological worry.* Journal of Psychiatric Research, 2009. **43**(6): p. 627-633.

6. Hayes, S., C.R. Hirsch, and A. Mathews, *Facilitating a benign attentional bias reduces negative thought intrusions.* Journal of Abnormal Psychology, 2010. **119**(1): p. 235-240.

7. Bar-Haim, Y., I. Morag, and S. Glickman, *Training anxious children to disengage attention from threat: a randomized controlled trial.* Journal of Child Psychology and Psychiatry, 2011. **52**(8): p. 861-869.

8. Li, S., et al., *Continual training of attentional bias in social anxiety.* Behaviour Research and Therapy, 2008. **46**(8): p. 905-912.

9. Amir, N., et al., *The effect of a single-session attention modification program on response to a public-speaking challenge in socially anxious individuals.* Journal of Abnormal Psychology, 2008. **117**(4): p. 860-868.

10. Browning, M., et al., *Using Attentional Bias Modification as a Cognitive Vaccine Against Depression.* Biological Psychiatry, 2012. **72**(7): p. 572-579.

11. Eldar, S., T. Ricon, and Y. Bar-Haim, *Plasticity in attention: Implications for stress response in children.* Behaviour Research and Therapy, 2008. **46**(4): p. 450-461.

12. Krebs, G., C.R. Hirsch, and A. Mathews, *The effect of attention modification with explicit vs. minimal instructions on worry.* Behaviour Research and Therapy, 2010. **48**(3): p. 251-256.

13. Hirsch, C.R., et al., *The contribution of attentional bias to worry: Distinguishing the roles of selective engagement and disengagement.* Journal of Anxiety Disorders, 2011. **25**(2): p. 272-277.

14. MacLeod, C., et al., *Selective attention and emotional vulnerability: Assessing the causal basis of their association through the experimental manipulation of attentional bias.* Journal of Abnormal Psychology, 2002. **111**(1): p. 107-123.

15. Dandeneau, S.D. and M.W. Baldwin, *The buffering effects of rejection-inhibiting attentional training on social and performance threat among adult students.* Contemporary Educational Psychology, 2009. **34**(1): p. 42-50.

16. Britton, J.C., et al., *Training-associated changes and stability of attention bias in youth: Implications for Attention Bias Modification Treatment for pediatric anxiety.* Developmental Cognitive Neuroscience, 2013. **4**: p. 52-64.

17. Schoorl, M., P. Putman, and W. Van Der Does, *Attentional Bias Modification in Posttraumatic Stress Disorder: A Randomized Controlled Trial.* Psychotherapy and Psychosomatics, 2013. **82**(2): p. 99-105.

18. Boettcher, J., et al., *Internet-Based Attention Bias Modification for Social Anxiety: A Randomised Controlled Comparison of Training towards Negative and Training Towards Positive Cues.* PLoS ONE, 2013. **8**(9): p. e71760.

19. Boettcher, J., T. Berger, and B. Renneberg, *Internet-Based Attention Training for Social Anxiety: A Randomized Controlled Trial.* Cognitive Therapy and Research, 2012. **36**(5): p. 522-536.

20. Carlbring, P., et al., *Internet-delivered attention bias modification training in individuals with social anxiety disorder - a double blind randomized controlled trial.* BMC Psychiatry, 2012. **12**(1): p. 66.

21. Neubauer, K., et al., *Internet-delivered attention modification training as a treatment for social phobia: A randomized controlled trial.* Behaviour Research and Therapy, 2013. **51**(2): p. 87-97.

22. Rapee, R.M., et al., *Integrating cognitive bias modification into a standard cognitive behavioural treatment package for social phobia: A randomized controlled trial.* Behaviour Research and Therapy, 2013. **51**(4–5): p. 207-215.

23. Kruijt, A.-W., P. Putman, and W. Van der Does, *The effects of a visual search attentional bias modification paradigm on attentional bias in dysphoric individuals.* Journal of Behavior Therapy and Experimental Psychiatry, 2013. **44**(2): p. 248-254.

24. Klumpp, H. and N. Amir, *Preliminary study of attention training to threat and neutral faces on anxious reactivity to a social stressor in social anxiety.* Cognitive Therapy and Research, 2010. **34**(3): p. 263-271.

25. White, L.K., et al., *Cascading effects: The influence of attention bias to threat on the interpretation of ambiguous information.* Behaviour Research and Therapy, 2011. **49**(4): p. 244-251.

26. Van Bockstaele, B., et al., *Effects of attention training on self-reported, implicit, physiological and behavioural measures of spider fear.* Journal of Behavior Therapy and Experimental Psychiatry, 2011. **42**(2): p. 211-218.

27. Najmi, S. and N. Amir, *The effect of attention training on a behavioral test of contamination fears in individuals with subclinical obsessive-compulsive symptoms.* Journal of Abnormal Psychology, 2010. **119**(1): p. 136-142.

28. Reese, H.E., et al., *Attention training for reducing spider fear in spider-fearful individuals.* Journal of Anxiety Disorders, 2010. **24**(7): p. 657-662.

29. Dandeneau, S.D., et al., *Cutting stress off at the pass: Reducing vigilance and responsiveness to social threat by manipulating attention.* Journal of Personality and Social Psychology, 2007. **93**(4): p. 651-666.

30. Bunnell, B.E., D.C. Beidel, and F. Mesa, *A Randomized Trial of Attention Training for Generalized Social Phobia: Does Attention Training Change Social Behavior?* Behavior Therapy, In press.

31. Dandeneau, S.D. and M.W. Baldwin, *The Inhibition Of Socially Rejecting Information Among People With High Versus Low Self-Esteem: The Role Of Attentional Bias And The Effects Of Bias Reduction Training.* Journal of Social and Clinical Psychology, 2004. **23**(4): p. 584-602.

32. Koster, E.H., et al., *Attentional retraining procedures: Manipulating early or late components of attentional bias?* Emotion, 2010. **10**(2): p. 230-236.

33. MacLeod, C., et al., *Internet-delivered assessment and manipulation of anxiety-linked attentional bias: Validation of a free-access attentional probe software package.* Behavior Research Methods, 2007. **39**(3): p. 533-538.

34. Eldar, S. and Y. Bar-Haim, *Neural plasticity in response to attention training in anxiety.* Psychological Medicine: A Journal of Research in Psychiatry and the Allied Sciences, 2010. **40**(4): p. 667-677.

35. Browning, M., et al., *Lateral prefrontal cortex mediates the cognitive modification of attentional bias.* Biological Psychiatry, 2010. **67**(10): p. 919-925.

36. Blaut, A., et al., *Are attentional bias and memory bias for negative words causally related?* Journal of Behavior Therapy and Experimental Psychiatry, 2013. **44**(3): p. 293-299.

37. Brosan, L., et al., *Cognitive bias modification for attention and interpretation reduces trait and state anxiety in anxious patients referred to an out-patient service: Results from a pilot study.* Journal of Behavior Therapy and Experimental Psychiatry, 2011. **42**(3): p. 258-264.

38. Taylor, C.T., J. Bomyea, and N. Amir, *Malleability of attentional bias for positive emotional information and anxiety vulnerability.* Emotion, 2011. **11**(1): p. 127-138.

39. Suway, J.G., et al., *Modification of threat-processing in non-anxious individuals: A preliminary behavioral and ERP study.* Journal of Behavior Therapy and Experimental Psychiatry, 2013. **44**(3): p. 285-292.
